# The Plague

**Published:** 1897-04

**Authors:** 


A Monthly Review of Medicine and Surgery.

*EDITORS:*

THOMAS LOTHROP, M. D. - - WM. WARREN POTTER, M. D.

All communications, whether of a literary or business nature, should be addressed to the managing editor: 284 FRANKLIN STREET, BUFFALO, N. Y.

VOL. XXXVI. APRIL, 1897. No. 9.

### THE PLAGUE.

THE reappearance and frightful devastation of life in India by the bubonic plague calls attention to one of the oldest-known and most abhorrent diseases afflicting the human family. Essentially a disease of *bad hygiene*, it has descended upon the people of those countries where cleanliness and sanitation were unknown and unthought of, and was consequently of frequent occurrence in Europe, Asia and Africa from the earliest writings up to the revival of learning in the sixteenth and seventeenth centuries. Especially prevalent was it during the fifteenth century in Northern Africa, Egypt, Arabia, Syria, Palestine, Asia Minor, Persia, India, China, and Europe generally. In the latter part of the seventeenth century a remarkable lessening of the area of prevalence of the disease began to take place. From 1660 to 1680, plague disappeared from Italy, England, Western Germany, Switzerland, Netherlands and Spain. During the next century but two serious outbreaks occurred in Europe, the first (1703–13) involving Turkey, Austria, Bohemia, Poland and Eastern Germany and the second (1720–22) involving Provence. At the close of the first third of the nineteenth century the area of prevalence in Europe was confined to the easternmost part of the Turkish Empire, and in 1841 plague ceased on the continent altogether. While this change was going on in Europe, the plague disappeared from Northern Africa (except Egypt), from Mesopotamia, Persia, and in 1844 in Asia Minor, Palestine and Syria. Since then it has been confined almost entirely, epidemically, in China and India especially, while sporadic limited outbreaks have taken place in Persia, Asia Minor and occasionally in Arabia

Several memorable outbreaks have taken place, especially the "black death," which spread over the whole of Europe and Asia in the fourteenth century, causing an inconceivable mortality ; the great plague of London, 1665 ; also the outbreak of 1780 in Marseilles ; the plague of Moscow, 1770, and the later outbreaks in Turkey, Syria and Egypt. These have become historical from the fatality which accompanied them. But still more memorable, from these terrible outbreaks, will remain the one which occurred in Hong Kong in May, 1894, when Kitasato discovered the true cause underlying the dreaded disease.

The bacillus discovered by Kitasato is a short rod with rounded ends, resembling the bacillus of chicken cholera, growing in a perfectly characteristic manner. His conclusions regarding the bacillus are as follows : (1) In the plague, bacilli are found in the blood, glands and viscera. (2) This particular bacillus is not found in any other disease. (3) Obtained in pure culture, it is capable of producing in inoculated animals the same effects as in human beings. (4) It gains entrance into the body through (*a*) the respiratory tract, (*b*) excoriations of the skin, (*c*) the digestive tract.

It may, therefore, be said that its cause, once discovered, therapeutic procedure will soon be advanced to cope with this most fatal disease, and these means have already been tried in the Bombay epidemic with considerable success.

